# Psychosocial Characteristics and Obstetric Health of Women Attending a Specialist Substance Use Antenatal Clinic in a Large Metropolitan Hospital

**DOI:** 10.1155/2011/729237

**Published:** 2011-05-02

**Authors:** Lucy Burns, Elizabeth Conroy, Elizabeth A. Moore, Delyse Hutchinson, Paul S. Haber

**Affiliations:** ^1^National Drug and Alcohol Research Centre, Faculty of Medicine, University of New South Wales, Sydney, NSW 2052, Australia; ^2^School of Psychiatry, Faculty of Medicine, University of New South Wales, Sydney NSW 2052, Australia; ^3^Schizophrenia Research Institute, Sydney NSW 2010, Australia; ^4^Royal Prince Alfred Hospital Drug Health Service, Sydney NSW 2050, Australia; ^5^Discipline of Addiction Medicine, University of Sydney, Sydney NSW 2006, Australia

## Abstract

*Objective*. This paper reports the findings comparing the obstetrical health, antenatal care, and psychosocial characteristics of pregnant women with a known history of substance dependence (*n* = 41) and a comparison group of pregnant women attending a general antenatal clinic (*n* = 47). *Method*. Face-to-face interviews were used to assess obstetrical health, antenatal care, physical and mental functioning, substance use, and exposure to violence. *Results*. The substance-dependent group had more difficulty accessing antenatal care and reported more obstetrical health complications during pregnancy. Women in the substance-dependent group were more likely to report not wanting to become pregnant and were less likely to report using birth control at the time of conception. *Conclusions*. The profile of pregnant women (in specialised antenatal care for substance dependence) is one of severe disadvantage and poor health. The challenge is to develop and resource innovative and effective multisectoral systems to educate women and provide effective care for both women and infants.

## 1. Introduction

There has been a wealth of literature examining the impact of substance use in pregnancy with findings consistently noting the association between maternal substance use and negative fetal outcomes including fetal respiratory distress, preterm delivery, small-for-gestational-age birth, and higher infant mortality in the first year of life [1–4]. Moreover, this association is complicated by the significant physical and psychosocial morbidity associated with substance use disorders. Studies have previously found high exposure to violence, poor physical health, and moderate/severe psychological distress among substance-using pregnant women [5–7]. It is often argued that integrated service delivery and intensive case management is needed to ensure the well-being of the mother-infant dyad throughout pregnancy and into the longer term. 

The health care system in Australia is largely divided into specialist streams and segregated levels of care. Such a system is often criticized for being too divisive and inadequate for the wholistic care of patients who present with multiple problems [8, 9]. In New South Wales, different models of integrated care for substance-using pregnant women exist. This includes perinatal consultation and liaison services operated from drug and alcohol treatment centres and multidisciplinary teams situated within antenatal clinics. 

This paper reports on a specialist antenatal clinic of a large public hospital in Sydney, where a collaborative approach to the care of pregnant women with known substance use problems has been adopted. The specialist clinic is conducted weekly from the Maternal and Child Health Department and is convened by staff from a number of different departments including Obstetrics and Gyneacology, Maternal and Child Health, Drug Health, and Social Work. The objective of the specialist clinic is to address the myriad of physical and psychosocial problems that substance using pregnant women present with in order to improve maternal and infant outcomes for the duration of the pregnancy and into the postpartum period. Referral to the specialist antenatal clinic can occur via a number of pathways including community-based general practitioners, the Drug Health Service, the Aboriginal Medical Service, and women attending other antenatal clinics who screen positive for substance use (all women are assessed for substance use, domestic violence exposure, nutritional status, and depression using a standardised psychosocial screening tool at their first booked appointment regardless of the antenatal clinic they attend).

The aim of the present study was to describe the clinical and psychosocial characteristics of pregnant women attending the specialist substance use antenatal clinic of a large public hospital in Sydney, Australia, and to compare these to the characteristics of a group of women attending a generalist antenatal clinic. This information was collected as a prerequisite to the design of a clinical service that could better meet the needs of both mother and child.

## 2. Materials and Method

The present study is a retrospective, cross-sectional study examining the characteristics of women with a known history of substance dependence accessing antenatal care from a clinic of a large public hospital in Sydney, Australia. A specialist clinic for women with substance use problems is run in conjunction with the general antenatal clinic and provides additional support around substance use and associated psychosocial issues. Women with a known history of substance use were approached to participate by the Drug Health Service Perinatal Clinical Nurse Consultant. All women attending the general antenatal clinic or birth centre of the same hospital were eligible to participate in the comparison group. Women in the comparison group were either invited to participate by a researcher while waiting for an appointment, or responded to advertisements posted in the clinic waiting room. Face-to-face interviews were conducted by researchers trained to administer structured clinical interviews, at either the antenatal clinic or the National Drug and Alcohol Research Centre, and took on average 1 hour to complete. All women provided written consent prior to participating. While no women were screened and deemed to be ineligible, a number of women declined to participate with the majority citing they were too busy or had too many other things going on. As such, it took approximately 18 months of recruitment to achieve the sample size reported. The study was approved by the University of New South Wales Human Research Ethics Committee (ref: 06254 and 08224) and the Ethics Review Committee (RPAH Zone) of the Sydney South West Area Health Service (ref: X06-0285 and 08/RPAH/218).

The interview included sections on the following: demographic characteristics, previous and current pregnancy characteristics, antenatal care, physical health service utilization, substance use history, injecting risk behaviour, exposure to violence, and mental and physical functioning. Health status was measured using the Short Form 12-item Health Survey (SF-12) [10]. The SF-12 generates two summary scores reflecting general physical and mental health functioning. The Depression Anxiety Stress Scale short version (DASS21) [11] was used to measure symptoms of depression, anxiety, and stress. The DASS21 has good reliability and validity and includes normative data for the Australian population. The Opiate Treatment Index (OTI) [12] was used to measure social functioning and the extent of substance use. The OTI provides an estimate of substance use based on the last three occasions of use. All other questions were derived from previous research conducted by the National Drug and Alcohol Research Centre (NDARC) (details can be provided by the authors on request).

## 3. Statistical Analyses

The data were analysed using PASW version 18 [13]. *t*-tests were used for continuous variables with means reported. Odds ratios and 95% confidence intervals were reported for nondichotomous categorical variables (OR, 95% CI) to determine if differences existed between substance dependent and comparison groups. There was no missing data for either sample.

## 4. Results

The results are based on interviews with 41 pregnant women with a known history of substance dependence (i.e., substance dependent group), and a comparison group of 47 women attending the general antenatal clinic (i.e., comparison group). Twenty-eight (70%) women in the substance dependent group were currently receiving substance use treatment, the majority (71%) of whom were enrolled in opioid pharmacotherapy (with a median duration of 11 months). 

Table 1 displays the demographic and psychosocial characteristics of the women. 

### 4.1. Antenatal Care

The women in the substance-dependent group were significantly more likely to present for their first antenatal visit later in their pregnancy than the comparison group (mean 14 versus 11 weeks pregnant, *t*_63_ = −3.02, *P* < .05). While this is still within the hospital's protocol for low-risk pregnancies (i.e., by 20 weeks gestation), the women in the substance dependent group would be considered high-risk, and just over a third (34%) of the women presented before 12 weeks, as per the hospital's protocol for high-risk pregnancies. A small proportion of women in the substance-dependent and comparison group (11% versus 2%, resp.) presented after 20 weeks gestation. The majority of the women in the substance-dependent group (76%) experienced some difficulty in accessing antenatal care (compared to only 20% of the comparison group): 40% (versus 15%) were unable to get an appointment; 24% (versus 0%) had “too many other things going on;” 17% (versus 0%) had insufficient money; 15% (versus 2%) had no transport to get the hospital clinic; 7% (versus 2%) could not get time off work; 12% (versus 0%) did not want others to know of their pregnancy; 10% (versus 0%) reported not having childcare available. None of the women reported difficulty accessing antenatal care due to lack of a Medicare card. All Australians are entitled to free ambulatory medical care upon presentation of their Medicare card, but if misplaced, procedures to access free care are more cumbersome.

### 4.2. Physical Health

Women in the substance-dependent group were more than 3 times more likely to report at least one obstetrical health complication during pregnancy (63% versus 34%, OR 3.36, 95% CI 1.40–8.07, *P* < .05). Severe vomiting and dehydration (54% versus 23%) was the most commonly reported health problem by women in both groups, followed by vaginal bleeding (27% versus 9%), problems with the placenta (12% versus 2%), and foetal growth retardation (12% versus 0%). Blood clots, kidney/bladder infection, and very high fever were each reported by 7% of the women in the treatment sample for each condition. Women in the comparison group were more likely to report high blood pressure (6% versus 2%). One woman in the substance-dependent group reported an incompetent cervix. None of the women in either group experienced gestational diabetes.

### 4.3. Obstetric Health

Most of the women (61%) in the substance-dependent group were in their third trimester of pregnancy at the time of being interviewed (mean 30 weeks; range: 11–40 weeks), whereas the comparison group were more likely to be in the second trimester when interviewed (mean 27 weeks; range 12–39 weeks). Seventy-five percent of the substance-dependent group and 57 percent of the comparison group had at least one prior pregnancy. Among these women, the substance-dependent group were significantly more likely to report a previous live birth (65% versus 43%, OR 2.49, 95% CI 1.03–6.06, *P* < .05) and report a previous termination (62% versus 19%, OR 6.94, 95% CI 2.59–18.57, *P* < .001), compared to the comparison group. Additionally, the women in the substance-dependent group were more likely to report a previous premature delivery (14% versus 6%), a previous cesarean section (11% versus 6%), and a previous miscarriage (46% versus 28%), compared to the comparison group, but these differences were not statistically significant. One woman in each group reported they had an infant who died within the first year. None of the women in either group reported a still birth.

### 4.4. Pregnancy Planning

While the majority of the women in the comparison group (79%) reported “wanting to become pregnant,” only 29% of the women in the substance-dependent group reported the same feelings (i.e., 9 times less likely (95% CI 3.39–23.81, *P* < .001). A substantial proportion of women in the comparison group reported they had not thought about becoming pregnant (49% versus 21%, OR 3.52, 95% CI 1.39–8.92, *P* < .05). Whilst 22% of the women in the substance dependent group specifically stated they had not wanted to become pregnant (compared to 0% in the comparison group). Of those who had not thought about being pregnant or did not want to become pregnant (29 women in the substance-dependent group and 10 women in the comparison group), only 14% of the substance-dependent group (versus 40% comparison group) reported using birth control at the time of conception.

### 4.5. Mental Health

The substance-dependent group had poor psychosocial functioning as evidenced by the low mean scores on the SF-12. The SF-12 is standardised to have a mean of 50 and a standard deviation of 10; thus, the average scores indicate that these women are more than half a standard deviation below the population norm for mental functioning and almost one standard deviation below the population norm for physical functioning. While the comparison group had a significantly higher SF-12 score for mental functioning, their physical functioning score was fairly comparable to the substance-dependent group. It is likely that the health effects of pregnancy account for below population norm scores on the physical functioning subscale for this group. 

The mean scores for the DASS subscales show the majority of women in the substance dependent and comparison groups scored in the normal/mild range for depression (81% versus 92%, resp.), anxiety (73% versus 89%, resp.), and stress (75% versus 94%, resp.). A higher proportion of women in the substance-dependent group, compared to the comparison reported high levels of distress (thus elevating the mean score for the sample): 15% (versus 0%) of the women were rated as severe/extremely severe for depression, 10% (versus 6%) as severe/extremely severe for stress, and 10% (versus 2%) as severe/extremely severe for anxiety. 

### 4.6. Substance Use

The mean age at first use of any substance was 14 years for the substance-dependent group (range: 9–20 years) and 13 years for the comparison group (range: 9–19 years). Alcohol was the drug first used by the majority of women in both the substance-dependent and comparison groups (46% versus 98%, resp.), followed by cannabis (37% versus 2%, resp.), and heroin (12% versus 0, resp.).  Table 2 shows the prevalence of lifetime and recent substance use. 

The women in comparison group reported no illicit drug use in the past month. Cannabis was the most prevalent illicit drug used in the past month by women in the substance-dependent group (34%). Among those women who used cannabis in the preceding month, the mean number of days used was 18, with most women using multiple times per day. Just under one fifth (17%) of the women had used heroin in the preceding month, on average once every five to six days. Benzodiazepine use was reported by 12% of the substance dependent group; however, the majority of the women reported one dose per day which possibly reflects prescribed use. With regard to licit drug use, women in the comparison group were significantly more likely to report alcohol use in the preceding month (49% versus 23%), with most women in both groups reporting just under 4 days of alcohol use during this period. An examination of individual OTI scores revealed no evidence of binge drinking in the past month. Seventy-eight percent of the women in the substance dependent group had smoked a cigarette in the preceding month (compared with 4% of the comparison group). While the women in the substance-dependent group reported smoking more days in the past month, the women in the comparison group reported smoking more cigarettes per day (mean 13 compared to 10). 

Twelve percent of the women in the substance-dependent group reported injecting heroin in the month preceding the interview, with the majority reporting this occurred once a week or less (95%). Of the women who reported injecting in the past month, half reported needle sharing on at least one occasion. Fifty-five percent of the women in the substance-dependent group had hepatitis C. The mean age at which they were first diagnosed with the disease was 22 years old. None of the women reported hepatitis B or HIV/AIDS infection.

### 4.7. Exposure to Violence

Women in the substance-dependent group were more than 3 times more likely to report being the victims of violence in the preceding twelve months, compared to the comparison group. Despite this, a small proportion of women in the comparison group reported being a victim of violence in the past year, but the context was very different to that of the substance-dependent group. The majority of women in the substance-dependent group reported the violence occurred multiple times within the year at home, and it was perpetrated by their partner. While in the comparison group, the incidences of violence reported were mostly isolated, occurring in the workplace; for example, the violence was perpetrated by a mental health patient towards a nurse.

## 5. Discussion

This study documents a broad spectrum of physical and psychosocial concerns among pregnant women with a known history of substance dependence attending a specialist antenatal clinic, and a comparison group of women attending the general antenatal clinic, of a large public hospital in Sydney. The findings indicate that while a comprehensive model of care has been implemented, this may still be inadequate to meet clinical needs of this group. The results suggest the profile of pregnant women (in specialised antenatal care for substance dependence) is one of severe disadvantage and poor health.

Many of the current pregnancies were unplanned, and there was low use of birth control practices among the women. A number of women commented that they did not think they could get pregnant. This may be related to the high rate of menstrual dysfunction among opioid-dependent females [14, 15] and suggests that there is a need for education regarding the importance of birth control even in the context of absent or irregular menses. In addition, cost-effective strategies to increase the use of contraception in this population need to be developed and implemented. This is a complex issue, as women with substance use problems may find the daily adherence to an oral or barrier method of contraception difficult, given their often chaotic lifestyle. 

Women in the substance-dependent group reported substantially higher rates of severe vomiting and dehydration compared to the comparison group. This difference may in part be explained by the fact that nausea and vomiting are direct actions of opioid drugs. While individuals stabilised on methadone with an established tolerance typically will not experience these adverse effects, an increase in dose (which may be required in pregnancy) can precipitate a recurrence [16, 17]. The majority of the women evidenced normal mental health as measured by the DASS21. This is somewhat surprising, given the high prevalence of psychiatric comorbidity consistently reported among substance use populations [18–21] but may be a reflection of the level of care the women received through this antenatal clinic. However, a proportion of the women in this study continued to report severe levels of psychological distress. This is of significant concern, given that psychological distress is associated with an increased risk for low birth weight and preterm delivery [2, 22] as well as an increased risk for maternal depression in the postpartum period [23]. 

Exposure to violence is also a significant concern for a proportion of the women in this sample, especially as partners were the predominant perpetrators of the violence for women in the substance-dependent group, and many of these women may continue in these relationships following the birth. This has a number of implications for the well-being of both mother and infant in the longer term, as the risk for a range of adverse outcomes, such as child maltreatment and homelessness, are strongly associated with family violence [24, 25]. Additionally, physical violence has been found to be an independent risk factor for low birth weight infants, possibly mediated by the impact of maternal stress on foetal development [26]. Studies examining violence, psychological stress, and depression show there is significant interplay between these factors, suggesting that concurrent management of these issues is warranted [5]. Almost all the women in the substance-dependent group were interviewed in their last trimester of pregnancy, and most had been attending the specialist antenatal clinic since early in their second trimester. The continued use of illicit drugs within this context is disquieting and underscores the chronic nature of substance dependence. 

Whilst the proportion of women who had used heroin in the past month was relatively low, most had used the drug regularly and almost all had injected it. Whilst pregnancy is commonly thought to be associated with positive changes in substance use in the general population, it may not have the same impetus among women with severe substance dependence. Why is it that these rates of drug use are reported among women attending a specialist antenatal clinic designed specifically for them? It is possible the women are reticent to disclose ongoing drug use to clinic staff for fear of notification to child protection authorities. This suggests that screening for substance use during pregnancy among women already receiving drug treatment may need to be reviewed. In particular, nicotine use has been associated with an increased risk for use of other substances [27] and could be seen as a flag for a more detailed inquiry of substance use throughout the pregnancy. This could be coupled with information and advice regarding the supportive role that child protection agencies and health service providers can have in ensuring the long-term well-being of both the women and their infants. 

In addition, the substantially high rates of nicotine use are of a concern considering the adverse health effects which are consistently identified for both mother and foetus including pregnancy complications and poor birth outcomes (e.g., placental abruption, low birthweight, neonatal death, and preterm birth [28, 29]. Women in the substance-dependent group were smoking on average 10 cigarettes per day (less than the comparison group), indicating they may been aware of such adverse effects and have tried to cut down but find it extremely difficult to quit. The findings imply the dependence severity of nicotine is severe, with research suggestive that the addictive properties of nicotine are comparable to other illicit drug types [30, 31]. In addition, a recent Cochrane review suggests alternative interventions (i.e., providing incentives through use of vouchers or financial rewards) should be considered [32]. This suggestion seems particularly salient among at risk groups, as it is likely that the high cost of nicotine-replacement therapies make them inaccessible to disadvantaged groups within the community. 

Of concern is the finding that just under half of the women in the comparison group reported alcohol use in the past month, with the majority reporting alcohol use on average once a week. Current Australian and international guidelines recommend that no alcohol during pregnancy is the safest option [33], as a threshold for negative effects on the neonate are yet to be determined. The results combined with anecdotal reports from the women that they received conflicting advice from doctors supports previous findings that there is a lack of knowledge about the risks to the foetus and uncertainty regarding safe levels of alcohol use in pregnancy [34, 35]. More research is required to ease uncertainty, and public health campaigns would assist in educating women about the risks of alcohol use in pregnancy. 

A number of limitations require mentioning. First, this study relied on retrospective self-report. Reluctance to answer sensitive questions such as substance use and exposure to violence may have resulted in an underestimate of these problems. Studies comparing urine toxicology and self-report measures of substance use have shown that this is likely for illicit but not licit substances [31]. Secondly, the study is based on a conservative sample of pregnant women who are engaged in health care and who consented to being interviewed. The recruitment process was difficult and protracted (approximately 18 months). It is possible that the more chaotic women declined to participate (possibly due to fears that child protection services would become involved), and therefore, the study findings are likely to be an underestimate of the extent of psychosocial and physical health problems associated with substance dependence in pregnancy. Finally, the study utilised a sample of pregnant women attending a specific substance use antenatal clinic in a large inner metropolitan hospital; the results, therefore, may not be applicable to all substance-dependent pregnant women. All women in the substance-dependent group had a history of problematic substance use, and it should be noted as a limitation that not all women were currently receiving pharmacotherapy treatments. This may have influenced some of the results related to mental health and antenatal care outcomes.

## 6. Conclusions

These limitations notwithstanding, the findings of the present study highlight the need to look beyond the neonatal impact of substance use during pregnancy and consider both neonatal and maternal indices of well-being. Maternal and infant health is interdependent; in order to achieve the best outcomes for infants, improvements in antenatal care are required to address the complexity of physical and psychosocial problems in the mother. In particular, there is a need to further develop comprehensive and integrated strategies that address (1) the complex interplay between interpersonal violence, psychological stress, low mood, and substance use, (2) the continued use of substances commonly perceived by substance-dependent persons to be less problematic (i.e., nicotine and cannabis), and (3) the poor understanding of female reproductive health and limited use of contraception. These health concerns are readily treatable. What is lacking is a coordinated approach that targets multiple problems within a single intervention. More specifically, the women reported difficulties in accessing health care suggesting that a more coordinated and flexible model of care may be warranted to both engage and maintain an effective line of care and treatment.

## Figures and Tables

**Table 1:** Demographic and psychosocial characteristics of pregnant substance dependent women and comparison group.

	Substance dependent group (*n* = 41)	Comparison group (*n* = 47)	Statistical comparison
Age (in years) mean (SD)	28.8 (5.3)	33.3 (4.2)	*t*_86_ = 4.47**
Years of completed schooling mean (SD)	10.3 (1.6)	11.9 (0.5)	*t*_46_ = 6.12**
% Currently married/de facto	46.3	93.6	OR 0.06 (95% CI 0.02–0.22)**
% ATSI	26.8	0	N/A
% Unemployed	80.5	21.3	OR 15.3 (5.4–43.2)**
% Currently homeless	12.2	2.1	not significant
% Exposed to violence in past year	24.4	8.5	OR 3.5 (1.00–12.08)*
SF-12 Mental functioning score mean (SD)	43.2 (12.7)	52.5 (7.3)	*t*_60_ = 4.1**
SF-12 Physical functioning score mean (SD)	41.4 (9.4)	44.7 (7.8)	not significant
DASS Depression score mean (SD)	9.9 (11.2)	4.4 (5.2)	*t*_55_ = −2.9*
DASS Anxiety score mean (SD)	6.7 (6.3)	3.0 (5.3)	*t*_77_ = −2.9*
DASS Stress score mean (SD)	12.5 (10.5)	7.7 (7.5)	*t*_69_ = −2.4*

*P < .05.

**P < .001.

**Table 2:** Lifetime and 1-month prevalence of substance use among substance-dependent women and comparison group.

		Substance dependent group (*n* = 41)	Comparison group (*n* = 47)	Statistical comparison OR (95% CI)
*ALCOHOL*	% Ever used	95.0	97.9	not significant
	% Used last month	22.5	48.9	0.30 (0.12–0.77)*
	Mean days used month (SD)	3.4 (2.9)	3.9 (2.3)	not significant

*NICOTINE*	% Ever used	97.6	74.5	13.70 (1.70–111.11)*
	% Used last month	78.0	4.3	80.0 (16.19–395.37)**
	Mean days used month (SD)	27.9 (6.8)	20.0 (14.1)	not significant

*CANNABIS*	% Ever used	95.1	83.0	not significant
	% Used last month	34.1	0.0	N/A
	Mean days used month (SD)	18.2 (12.6)	0	N/A

*HEROIN*	% Ever used	75.6	4.3	71.43 (14.29–333.33)**
	% Used last month	17.1	0.0	N/A
	Mean days used month (SD)	5.3 (6.9)	0	N/A
	% Ever injected	56.1	0.0	N/A
	% Injected last month	12.2	0.0	N/A

*AMPHETAMINE*	% Ever used	80.0	46.8	4.55 (1.73–11.90)*
	% Used last month	2.5	0.0	N/A
	Mean days used month (SD)	2.0 (0.3)	0	N/A
	% Ever injected	47.5	0.0	N/A
	% Injected last month	0.0	0.0	N/A

*COCAINE*	% Ever used	68.3	40.4	3.17 (1.32–7.63)*
	% Used last month	4.9	0.0	N/A
	Mean days used month (SD)	1.5 (0.7)	0	N/A
	% Ever injected	41.5	0.0	N/A
	% Injected last month	0.0	0.0	N/A

*BENZODIAZEPINE *	% Ever used	75.6	14.9	17.86 (6.06–52.63)**
	% Used last month	12.2	0.0	N/A
	Mean days used month (SD)	13.6 (15.0)	0	N/A
	% Ever injected	7.3	0.0	N/A
	% Injected last month	0.0	0.0	N/A

*P < .05.

**P < .001.
